# The autistic brain in the context of normal neurodevelopment

**DOI:** 10.3389/fnana.2015.00115

**Published:** 2015-08-25

**Authors:** Mark N. Ziats, Catherine Edmonson, Owen M. Rennert

**Affiliations:** ^1^National Institute of Child Health and Human Development, NIHBethesda, MD, USA; ^2^Medical Scientist Training Program, Baylor College of MedicineHouston, TX, USA; ^3^College of Medicine, University of FloridaGainesville, FL, USA

**Keywords:** autistic disorder, autism spectrum disorder, neurodevelopment, microglia, mini-columns, neural networks

## Abstract

The etiology of autism spectrum disorders (ASDs) is complex and largely unclear. Among various lines of inquiry, many have suggested convergence onto disruptions in both neural circuitry and immune regulation/glial cell function pathways. However, the interpretation of the relationship between these two putative mechanisms has largely focused on the role of exogenous factors and insults, such as maternal infection, in activating immune pathways that in turn result in neural network abnormalities. Yet, given recent insights into our understanding of human neurodevelopment, and in particular the critical role of glia and the immune system in *normal* brain development, it is important to consider these putative pathological processes in their appropriate normal neurodevelopmental context. In this review, we explore the hypothesis that the autistic brain cellular phenotype likely represents intrinsic abnormalities of glial/immune processes constitutively operant in normal brain development that result in the observed neural network dysfunction. We review recent studies demonstrating the intercalated role of neural circuit development, the immune system, and glial cells in the normal developing brain, and integrate them with studies demonstrating pathological alterations in these processes in autism. By discussing known abnormalities in the autistic brain in the context of normal brain development, we explore the hypothesis that the glial/immune component of ASD may instead be related to intrinsic exaggerated/abnormal constitutive neurodevelopmental processes such as network pruning. Moreover, this hypothesis may be relevant to other neurodevelopmental disorders that share genetic, pathologic, and clinical features with autism.

## Introduction

The complex processes that lead to the fully formed human brain encompass a spectrum of mechanisms spanning genetic determinates to environmental and experiential influences. While the specific mechanisms underlying human disorders of neurodevelopment, such as autism spectrum disorder (ASD), remain poorly understood, over the past several decades significant advances have been made to document the cellular and anatomical events that occur as the normal human brain develops and matures. It is therefore important to consider studies of autism in the context of normal cellular/anatomic brain developmental patterns, as it is likely that abnormalities in the autistic brain represent an over-exaggeration and/or under-utilization of normal physiological processes that are constitutively operant in neurodevelopment. This hypothesis is particularly relevant to studies of cytokines, the immune system, and glia in autistic patients, as abnormalities in these processes are often thought of as reaction to exogenous insults, yet it is entirely plausible instead that these findings represent aberrations of otherwise normal neurodevelopmental mechanisms. In this review, we explore this hypothesis by integrating what is known about normal human neurodevelopment with recent work suggesting immune and glial abnormalities may play a role in the development of autism.

## Discussion

Cellular human brain development is a protracted process that begins around the third post-conception week (pcw) and arguably extends nearly into adulthood (Stiles and Jernigan, 2010). Conventionally, human brain development is considered in gross stages within which major cellular and anatomic transitions occur: namely the embryonic, fetal, early and late postnatal, adolescent, and adult periods (Insel, 2010).

### Embryonic Period

Beginning early in the embryonic period (defined as conception to eight pcw), the basic structures of the brain, spinal cord, and peripheral nervous system are established. The first major differentiating event of the embryonic period is gastrulation, during which the single-layered blastula forms a trilaminar structure containing the ectoderm, mesoderm, and endoderm. Gastrulation is completed by the third pcw, at which time some cells of the ectodermal layer differentiate into neural progenitors (Ozair et al., 2013). The first well-defined neural structure, the neural tube, begins forming during the third pcw and serves as the basis of the early developing central nervous system (CNS), within which reside populations of neural stem cells. From this basic tubular structure, more specific neural patterning of what will become the major brain structures and compartments occurs through the creation and migration of neural cells from the stem cell proliferative zones. Through graded patterns of molecular signaling, neural progenitors migrate outward from proliferative zones and begin differentiation such that a primitive map of the brain is established by the end of the embryonic period. For instance, through comparative studies of other mammals it has been suggested that the sensimotor regions of the neocortex, the major compartments of the diencephalon and midbrain, and the organization of the hindbrain and spinal column are all well established by the end of the embryonic period in humans (Lumsden and Keynes, 1989; Bishop et al., 2002; Gavalas et al., 2003; Kiecker and Lumsden, 2004; Nakamura et al., 2005).

### Fetal Period

Around the ninth pcw, the fetal period of development ensues and extends until birth, during which time there is rapid growth of the structures established during the embryonic period. Grossly, the brain develops its characteristic gyri and sulci during the fetal period, reflecting the underlying dramatic cellular changes occurring during this period (Chi et al., 1977). The majority of neuronal and glial proliferation occurs between the 9th and 16th pcw, with the peak period of migration of these cells to their appropriate region following closely thereafter (Volpe, 2000). In fact, production of new neurons is largely finished by mid-gestation, except for the ongoing production of neurons in a few specialized areas (Bystron et al., 2008).

After their production in the proliferative regions, neurons migrate in an orderly manner to their final position in the developing brain. In the neocortex, the arriving cells establish a 6-layered structure, with the earlier migrating neurons forming the deeper layers and the later migrating neurons forming the more superficial layers (Cooper, 2008). Their migration from the proliferative zone to their final position in the neocortex is helped by the guidance of radial glial cells, a population of stem cells that serve as a scaffold in the developing brain of all vertebrates (Borrell and Götz, 2014). Different layers of the neocortex contain different types of neurons as a result of both cell-intrinsic mechanisms operant in the progenitor cells from which they derive, and through soluble signaling cascades that direct progenitors toward a restricted mature neuronal type (Desai and McConnell, 2000; Leone et al., 2008).

### Transient Structures

Of particular note in this migration process are a set of structures that appear only transiently during the fetal period to help guide the migration of progenitors to the developing neocortical layers. The very first neurons to populate the developing neocortex form a primitive and transient structure termed the preplate, which is then split into two separate structures by arriving neurons—the marginal zone and the subplate (Molnár et al., 2006). The region between the marginal zone and subplate serves as a hub for new arriving neurons, and will eventually become layer 6 (the deepest) of the developing neocortex. Subsequently, all newly arriving cells will form progressively more superficial layers of the neocortex from this base structure.

Intriguingly, both the marginal zone and subplate have been shown to highly express some of the genes most significantly linked to neurodevelopmental disorders such as autism and schizophrenia, such as *Reelin* and *TBR1* (Hevner et al., 2001; Bielle et al., 2005; Hoerder-Suabedissen et al., 2013). Specifically, studies have shown that a complete loss of *Reelin* or *TBR1* in post-mortem mouse brains results in severe disruption of subplate and marginal zone formation, leading to significant deficits in early-born cortical neuronal differentiation, migration, and axonal generation, ultimately resulting in a loss of regional identity (Hevner et al., 2001; Rice and Curran, 2001; Bedogni et al., 2010). While complete knockout of these genes may not be present in most ASD cases, it is plausible that smaller changes affecting *Reelin* and *TBR1*, such as copy number variations or single nucleotide variants, may cause changes in their expression resulting in subtle changes in cortical organization, and therefore contribute to the abnormalities in neocortical connectivity thought to underlie much of ASD pathology. While the marginal zone and subplate are clearly instrumental in the proper migration and formation of neurons to form mature neocortical networks, the transient nature of this structure during development makes it impossible to study in human post-mortem brain assessments of ASD patients. Further work to define the role of this structure in animal models of ASD will be important to explore the contribution these largely understudied structures may have to neurodevelopmental disorders.

### Cortical Mini-Columns

Similar to the unique structure and function of the marginal zone and subplate, cortical mini-columns and their formation are likely to be integral to the ASD phenotype. During the process of neuronal migration in the normal brain, developing neurons migrate from the germinal zones to predetermined areas of the neocortex and form mini-columns, the basic organizational unit of neuronal circuitry within the cortex (Rakic, 1988). Each mini-column is composed of 60–100 neurons, all with apical dendrites, myelinated, and double-bouquet axons (Mountcastle, 1997). Groups of mini-columns are organized into radial structures to form macro-columns, which then combine to make large networks that span layers II through VI of the neocortex (Mountcastle, 1978). The precise arrangement of neurons within these mini-columns is essential to cortical development, as it has been shown that even subtle alterations in the spacing of these mini-columns can alter the processing of information and overall circuitry of the neocortex (Seldon, 1981).

Intriguingly, post-mortem autistic brain tissue has been shown to have mini-columns that are narrower and contain more neurons than control brains, and additionally, the neurons within the autistic mini-columns are more dispersed (Casanova et al., 2002). Moreover, the abnormalities in mini-column structure that the authors observed in this study were most apparent in areas where GABA-ergic inhibitory interneurons predominated, suggesting lateral inhibition may be disrupted in autism brains (Marin-Padilla, 1970; DeFelipe and Jones, 1985; Casanova et al., 2002). This is of particular interest as many other separate investigations have provided support for global GABA-ergic dysfunction in ASD (Palmen et al., 2004; Voineagu et al., 2011). Specifically, aberrant GABA-ergic signaling in ASD is thought to create a more hyper-excitable state and result in deficits of filtering capacity and information processing within the cortex (Casanova et al., 2003; Rubenstein and Merzendich, 2003). It has been proposed that this deficit in inhibitory function may explain in part some for the behavioral phenotype of autism and the higher prevalence of seizures in ASD patients (Casanova et al., 2003; Brooks-Kayal, 2010).

As with the pre-plate and subplate, the migration of neurons into the developing mini-columns is impossible to study in human brain tissue, and can only be assessed after the completion of this process in post-mortem tissue from autistic patients. It is therefore impossible to determine whether the migration into mini-columns is aberrant, or if their migration is normal but the patterning and wiring of these newly arrived neurons is aberrant after they arrive in autistic patients. Animal and perhaps cellular models such as induced pluripotent stem cells (iPSCs) again will be important in helping to discern these possibilities, although it is entirely possible that both mechanisms are abnormal. In fact, there is a large body of evidence showing that fully-migrated neurons abnormally join neural networks and these networks are abnormally pruned in autistic brains, as is discussed next.

### Microglia and Synaptic Pruning

After the process of neuronal migration, young neurons begin to be incorporated into newly developing neural networks through a dynamic process of synaptogenesis and pruning that continues late into adolescence. Young neurons initially develop processes (dendrites and axons) that allow them to form synapses with other neurons both locally and long-distance. The growth cone of an axon is able to sample the neuron’s environment for both chemical and electrical signals that guide its wiring to other neurons to create a new synapse (Brown et al., 2002). Initial patterns of connectivity in the fetal and early postnatal brain are characterized by exuberant synaptic connections that will later be pruned away to leave only the connections indicated through postnatal experience (Stiles and Jernigan, 2010; Kettenmann et al., 2013). This process of network refinement occurs through both synaptic rewiring and neuronal apoptosis, with rates of apoptosis as high as 70% of cells in some regions of the cortex (Rabinowicz et al., 1996). Physiological neuronal apoptosis in development occurs both as the result of intrinsic neuronal cell death mechanisms mainly responding to the absence of local neurotrophic factors, and also through glial-initiated mechanisms which have recently become more widely recognized (Marín-Teva et al., 2004; Takahashi et al., 2005; Bessis et al., 2007).

While most research in the glial contribution to neurodevelopment has focused specifically on astrocytes, microglia—the resident immune cells of the CNS—were recently demonstrated to be involved in many fundamental neurodevelopmental processes including directing the invading vasculature and removing apoptotic cells (Parnaik et al., 2000; Streit, 2001; Stevens et al., 2007; Calderó et al., 2009; Paolicelli et al., 2011). Importantly, Paolicelli et al. (2011) demonstrated that microglial pruning of developing synapses is an absolute requirement for normal brain development. Moreover, others have shown that microglial cells in the developing cerebral cortex of prenatal and postnatal macaques and rats limit the production of cortical neurons by phagocytizing neural precursor cells as neurogenesis nears completion (Cunningham et al., 2013). Furthermore, studies of mice with abnormal numbers of microglia have shown that an alteration in microglial number perturbs neural development by directly affecting embryonic neural precursors and inducing astrogliosis (Antony et al., 2011). There is even evidence that microglia contribute to network remodeling in response to learning and stimulation (Dong and Greenough, 2004).

Despite these recent findings, the number of studies assessing the role of microglia in autistic brain tissue directly is strikingly small. Pardo et al. (2005) initially demonstrated that post-mortem brain tissue from patients with autism exhibits an increased microglial density in gray matter. Moreover, it was shown that microglia from MeCP2-null mice—a model of Rett Syndrome—produced a conditioned media that damaged synaptic connectivity via a glutamate-excitotoxicity mechanism (Stevens et al., 2007). Importantly, this effect was not seen in MeCP2-null astrocytes from the same animals, suggesting aberrant microglial activation during development may lead to improper brain development independent of other glial populations. Additionally, it was shown that microglia in autistic brain samples display an activated morphology and secrete a cytokine profile consistent with a pro-inflammatory state (Bailey et al., 1995). Furthermore, we recently demonstrated abnormal expression of both microglial- and astrocyte-specific cell markers in two regions of post-mortem autistic brain tissue (Edmonson et al., 2014). These findings are interesting as the immune response in the CNS of patients with ASD has received a considerable amount of attention since autism was first described. However, most theories suggest either an exogenous factor stimulating neuro-inflammation or an autoimmune activation in the CNS. Yet, as the contribution of microglia to proper neuronal network formation—independent of their immune function—becomes increasingly recognized, it is important to consider that the intrinsic developmental component of microglia may be perturbed in ASD, such that their role in construction of neural networks is abnormal independent of inflammatory reaction (Garbett et al., 2008). This is in contrast to the traditional view that an exogenous immune response causes neural network destruction, although again it is possible that both mechanisms may contribute to the ASD phenotype. We suggest, however, that the former has received far less attention in the ASD literature and a more thorough assessment of this hypothesis may help significantly reconcile the disparate findings of neural network dysfunction and immune/glial abnormalities in ASD.

### Postnatal Development

By the end of fetal development, all major adult brain structures are present, major connections between them are established, and the brain is poised for the rapid and dynamic growth that occurs in the first few years of life. The brain develops rapidly in the first few years after birth, reaching almost adult volume by age six (Lenroot and Giedd, 2006). While the production and migration of neurons are mainly prenatal events (with the notable exception of subventricular zone), glial progenitors have been shown to proliferate and differentiate throughout childhood, likely helping to sculpt the developing synaptic networks (Cayre et al., 2009).

One main function of proliferating glial cells during the early and late postnatal periods is to accomplish the extensive amount of axon myelination that occurs during this time. Increased myelination of axons allows for increased growth of axon diameter, and ultimately enables faster and long-distance neuronal connections (Zalc et al., 2008). Robust increases in myelination have been reported across the brain from ages 5–12 years, with a varying rate of fiber tract myelination in various brain regions (Lebel et al., 2008; Lebel and Beaulieu, 2009). These findings led to the thought that aberrant myelination may be contributing to ASD pathology, and contributed to the recent trend in the field towards diffusion tensor imaging (DTI) and functional magnetic resonance imaging (fMRI) studies. Kleinhans et al. (2012) reported white matter microstructure changes in multiple tracts of ASD brains across postnatal development as compared to control brains using DTI. Specifically, they reported decreased fractional anisotropy with increased radial diffusivity in all tracts except the brainstem, hypothesizing that these observed changes may reflect an underlying defect in long distance myelin tracts in ASD (Kleinhans et al., 2012). These findings support a previous report that showed post-mortem autistic brains had a decreased number of long distance axons in the white matter, and additionally, that the axons in autistic brains had increased branching and thinner myelin when compared to controls (Zikopoulos and Barbas, 2010). Integrating these observations with studies supporting increased local synaptic excitation in ASD, many have hypothesized that abnormalities in the autism brain are a consequence of increased local neocortical connectivity and decreased cortical inter-region connectivity (Courchesne and Pierce, 2005; Casanova et al., 2006; Casanova and Tillquist, 2008).

Finally, while much of the organization of the postnatal brain is genetically determined, it has been clearly demonstrated that this intrinsic development remains extremely malleable to experience-dependent processes (Hubel and Wiesel, 1977; Markham and Greenough, 2004; Stiles and Jernigan, 2010). Moreover, epigenetic mechanisms that ultimately converge to influence gene expression have been shown to be one of the main mediators between environmental experiences and developmental synaptic plasticity (Fagiolini et al., 2009). For instance, studies in mice have shown that environmental enrichment results in increased chromatin remodeling that modifies gene expression patterns in the hippocampus, resulting in improved spatial memory (Fischer et al., 2007). Alternatively, an increase in methylation of the *BDNF* promoter and consequent decrease in *BDNF* mRNA in the prefrontal cortex was found in association with exposure to periods of abusive maternal care, and these effects are perpetuated to the F1 generation suggesting a role for trans-generational effects (Champagne, 2008). Similarly, environmental insults to the developing brain, such as in fetal alcohol syndrome, have been shown to effect glial cells and their subsequent ability to effectively modulate neuronal development (Guizzetti et al., 2014). Yet, while studies of model organisms are beginning to demonstrate that gene expression represents a critical nexus of experience dependent plasticity, human studies of neurodevelopmental disorders in which this process may go awry are limited, and the general landscape of gene expression in the developing human brain as relates to neurodevelopmental disorders like autism is largely unexplored in relation to their corresponding cellular and network level changes. Integrating these scales of evidence will be an important future endeavor for the field to begin to truly integrate environmental/experiential influences with intrinsic mechanisms that ultimately shape neural network development and their aberration formation in ASD.

## Conclusion

In summary, great progress in understanding the anatomical and cellular trends underlying human brain development have been made over the past few decades. We have come to appreciate though various approaches that human neurodevelopment is a dynamic and protracted process, characterized by an initial period of neurogenesis leading to the formation of the basic CNS framework in early embryonic development. This is followed by substantial cellular proliferation, migration, and differentiation in the fetal period that establishes the main areas and pathways of the brain by birth. The early postnatal period is a time of rapid growth through glial proliferation, myelination, and organization of developing neural networks. Importantly, this process is very malleable particularly with regard to environmental and experiential events. Precise refinement of these developing neural networks occurs throughout adolescence and into early adulthood.

Previous research has led to the notion that ASD represent a complex interplay between the genome, immune signaling, and synaptic wiring; specifically, many of these studies have focused on the consequences of exogenous events on already developed neural networks. However, another potential explanation for these findings is that they represent an exaggeration or abnormality of normal processes that occur during the completion of brain development. Here, we propose that exaggerated and/or underutilized glial processes may be contributing to the construction of aberrant neural networks, apart from their more well-recognized immune functions. Moreover, as the genetic, pathologic, and clinical features of other neurobehavioral diseases like schizophrenia overlap considerably with ASD, it is possible that this hypothesis linking known immune and glial cell dysfunction in autism to aberrations in normal processes of neurogenesis may be broadly relevant to other neurodevelopmental disorders (Burbach and van der Zwaag, 2009; Tuchman et al., 2010; Mitchell, 2011). For instance, a large body of evidence has demonstrated glial abnormalities in both post-mortem human brain and animal models of Fragile × Syndrome (the most common single-gene disorder with autism as a component) and in Down Syndrome (Goodison et al., 1993; Greco et al., 2006; Jacobs and Doering, 2010). Future work should concentrate on further understanding precisely how these normal neurodevelopmental mechanisms go awry in autism and in related disorders, and how we can use these findings to develop biomarkers to help diagnose and ultimately treat neurodevelopmental disorders.

## Funding

This work was supported by the Intramural Research Program (IRP) of the National Institute of Child Health and Human Development, NIH. MNZ was also supported by the NIH-Cambridge Biomedical Scholars Program, and the Baylor College of Medicine Medical Scientist Training Program.

## Conflict of Interest Statement

The authors declare that the research was conducted in the absence of any commercial or financial relationships that could be construed as a potential conflict of interest.
